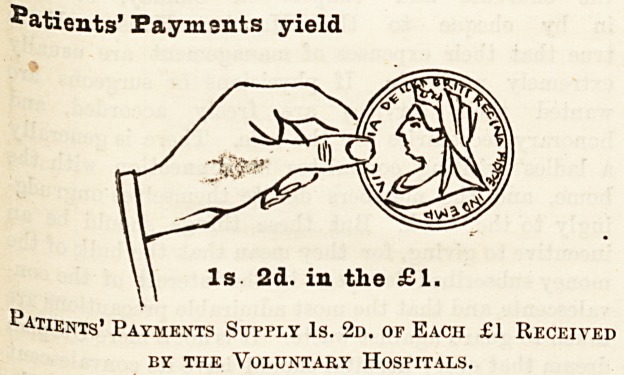# Special Hospital Sunday Supplement

**Published:** 1900-06-23

**Authors:** 


### Special Hospital Sunday Supplement.

### Besieged in the City of Pain.

Besieged! The meaning of that word has become very real to England since last October. Ladysmith, Wepener, Mafeking—each name has its own story. There are many hearts that ache over again with the aching of loneliness when one of these names is mentioned. There are many minds full of memories of opening each day's paper with an expectancy and a dread. There are men and women and little children all over the world to-day who thank God for, and are proud of, one of their family who helped "to keep the flag flying" during the siege. Yes! we of this generation have learned by sorrow, and by anxiety, and by pride, what a word almost forgotten means. And when London realised the meaning of a siege when the country was at war, London was stirred to the depths. For war meant sorrow and suffering. War meant pain. War meant disease, sickness, and death. War meant that the roll of "the fatherless children and widows" was increasing in length.

Then London realising all this, London translated its emotion into action. The Lord Mayor of London opened the Mansion House Fund for the sufferers from the war. Londoners rallied round him. Rich and poor, gentle and simple, did their best. Men, women, and little children sent their gifts of money to the War Fund. And each felt glad that they were privileged to do something, great or small, towards the relief of suffering and sorrow.

Besieged! There is a siege going on in London for 365 days each year. There is an army of 7,000 men, women, and children shut up in the hospitals of London every day. The garrison is beleaguered. The enemy rests not day or night. The sentries in the watch-towers oft grow weary; but can never rest. Sap and countersap, sap and countersap! That is the story of the fight going on every day in the hospitals of London against disease. Seven thousand men, women, and children shut up day after day! What suffering is theirs! What patience they need! What bravery they exhibit! Can nothing be done to relieve them?

This army is formed of conscripts. There is none of the glow of enthusiasm, of devotion to country, of loyalty to Queen, animating the breasts of the soldiers shut up in the City of Pain. There is no escape. The lot falls. A man here, a woman there, a child elsewhere, must silently lay aside the pursuits of ordinary life and fall into the ranks—ranks often overcrowded, but never full.

Walk down the wards of any of our London hospitals and scan the faces of the men, women, and children in this army of sufferers. There is a man upon whose face a look other than that of physical pain is imprinted. The physical pain is hard to bear; but this other is harder. The house physician tells us that look comes from mental anxiety, and that if only he could relieve that he could more hopefully resist the attacks of the enemy. That man is a clerk in a big City firm. He has a wife and children. The young couple were just beginning to "get straight" when the lot fell: rheumatic fever. The home has to be left. The conscript joins the ranks. But there is no pay given. Will the place in the City be kept open? The sufferer's heart aches with anxiety for wife and bairns. The prisoners in the London hospitals are in worse case than the prisoners were at Pretoria. Can nothing be done to relieve them?

The Hospital Sunday Fund gives the answer to both questions. Over it, as over the other War Fund, the Lord Mayor presides. The headquarters of both are at the Mansion House. The collections in every place of worship on June 24th mean that London wants to send relief to the suffering shut up in the City of Pain. To this fund rich and poor, gentle and simple, can also send their gifts. Then the cheering message can be borne to the brave men and women who are conducting the defence that London thinks of, and cares for, them and their work quite as much as for the men and women in South Africa.

Hospital Sunday this year, if London realises its privileges, ought to be the glad recognition by the people of this great metropolis of the existence of heroes in their midst; for truly the medical men and nurses in the London hospitals are doing heroic work. The devotion to duty, the pluck and endurance of fatigue, the weariness, the anxiety, the watchfulness—each and all of these qualities are exhibited in the hospitals of London every day. The talented consultant who is sought by the wealthy of the land, the gentle nurse whose patience seems inexhaustible, the strong young house physician or surgeon, seem to vie with each other in their resolve to "keep the flag flying," to resist the unceasing assaults of disease; to tell the enemy that, though the army of sufferers in the London hospitals be imprisoned in the city of pain, the great strong bonds of sympathy and love unite the officers and the rank and file in a common cause and a united determination—no surrender.

Come, then, you who dwell in this mighty metropolis of London; you who know how to praise and sympathise with courage, endurance, heroism; you who love to relieve suffering and sorrow. Let your contribution on Hospital Sunday be a measure of your knowledge of facts and of your appreciation of that which is noble and great. The army of 7,000 sufferers pleads for your help.

Sunday—Hospital Sunday—June 24th. The Lord's Day—the Lord Who "went about doing good;" Who said to His followers, "Heal the sick." This year it falls on the commemoration of the nativity of St. John Baptist. Need we further point the connection? The Baptist came "to prepare the way" for One Whose mission, He said, was "to preach the Gospel to the poor, to heal the broken-hearted, to preach deliverance to the captives." Hospital Sunday translates that mission into a practical reality for Londoners to-day; for "it takes a man to save a man."

### Hospital Revenues and the War Funds.

Every preacher and every thoughtful Londoner should put his heart into the effort to make Hospital Sunday in the metropolis this year a record. Both may welcome a reminder that ever since last November hospital managers have been crippled in their efforts to raise funds for the suffering and the sick. Those efforts have been crippled, not because money might not have been raised by extra effort on the part of the public for the hospitals, but for the reason that the metropolitan hospitals as a whole are most intelligently administered at the present time by patriotic men, who have had the courage for the most part to hold back their appeals pending the termination of the war. It must be recognised that the war in South Africa, and everything connected with it, has been the one predominant object in the public mind for the last seven months. It is only natural, therefore, that everybody should have thought first of all of the war funds, of the widows and orphans, and of the wounded and disabled soldiers and sailors, as well as of the wives and families left behind to the care of the nation. The widespread interest caused by the war in this country has temporarily affected everything, including the business of the country, and the same thing happened in the United States at the outbreak of the war with Spain. Fortunately, on the eve of Hospital Sunday the occupation of Pretoria by the British troops seems to indicate that the war in South Africa is approaching its termination. In such circumstances the sympathy of everybody who values the hospitals, or takes an intelligent interest in the maintenance of the great voluntary medical charities of the metropolis of the Empire, must go out to the needs of the hospitals, and stimulate them to strain every effort to so increase the amount collected on Hospital Sunday, 1900 (June 24th), as to recoup a substantial proportion of the loss experienced by these institutions during the last seven anxious months, owing to the claims of the war and of the funds connected with it, which have engaged the attention of the public.

Soon after the commencement of the war the *British Medical Journal* published some articles on this subject, in which confidence was expressed that almost without exception the hospitals would not venture to urge that they were receiving no contributions from the public owing to the war funds. It was maintained with confidence, and has proved to be accurate, that the metropolitan voluntary hospitals, being well administered, so organised their system of raising money that, in ordinary times, with the exception of a few of the summer months, money comes in to the hospital exchequer throughout the whole year. Each voluntary hospital that is well administered relies upon a double harvest during each period of twelve months. The first is reaped before Easter, the second becomes ripe with the commencement of October, and should be safely garnered within a week of Christmas Day. In such circumstances we have maintained that any hospital which is in want of funds to a large extent at the end of the month of November is not administered with sufficient energy and intelligence so far as its financial arrangements are concerned. Owing to the war funds—and this is the point to drive home—the best hospitals have withheld their appeals from motives of patriotism, and are consequently without the Easter offerings, a material fact in itself, and one which should appeal with extra force to all Christian people. Surely the self-denial of the hospital managers of London should win for them the active support and energetic co-operation of thousands of good people who have been working so zealously for the war funds, and the large majority of whom are probably regular attendants at places of worship. They have it in their power, and we gladly credit them with the will, if their attention can only be directed to the point, to set to work in co-operation with the clergy and ministers of religion throughout London to see that the sum raised on Hospital Sunday in every church and chapel shall be something like double the sum which has heretofore been collected on that day.

If the metropolitan daily Press, the editors of which have co-operated over and over again with The Hospital in directing public attention to the needs of the hospitals at this season in connection with the Sunday Fund, will kindly do their part as Londoners, grateful for the victory of the British arms, then there is no doubt that the lost Easter harvest will be recouped to the hospitals through the Hospital Sunday Fund. May we suggest to our contemporaries in the Press that the object all must have in view would be best secured by the devotion of a small amount of space each day to a few aspects of the hospitals' needs and claims in the week commencing June 18th? We would remind them as an encouragement that in 1895, when the lay press of the metropolis generously co-operated with this journal, upwards of £60,000 was raised on Hospital Sunday, and there is no reason, quite the contrary, why £100,000 should not be given as a free gift to the hospitals in connection with Hospital Sunday, 1900. The Lord Mayor has always received contributions for the hospitals in connection with the Sunday Fund at the Mansion House from those who, from absence from London or other causes, are unable to attend a place of worship. There must be thousands of families in the metropolis who have friends at the seat of war who would gladly welcome the occasion of Hospital Sunday in the circumstances we have here brought out as a means to enable them to give a substantial thank-offering for the escape of their loved ones from the perils of war, or for their recovery from wounds or sickness contracted in connection with the military operations in South Africa. In any case, whatever the result, in this hour of the Empire's triumph we appeal to the manhood of London of all classes, and especially to that of our colleagues in the lay press, to spare no effort to make the Hospital Sunday contributions to the hospitals worthy of the year and the occasion, and still more worthy of the Empire's metropolis, the greatest city in the world, where the pressing claims of the sick and suffering, owing to the terrible aggregate, are greater and more urgent than those of any other urban population.

### Our Soldiers and Our Hospitals.

The thoughts of the nation have been of late so centred in the doings of our soldiers in South Africa, that Hospital Sunday, with all that it connotes—the relief of suffering, the cure of the sick, the investigation of disease, and the expansion of medical knowledge—is likely to impress itself less forcibly upon the public mind than has been the case in quieter times, and in years less crowded with events of all-absorbing interest. Yet, if one takes broad views of things, one sees how intimately that aspect of the campaign which has elicited the most unstinted praise, namely, the care of the wounded, is connected with the daily work of our great hospitals, and how its success is the direct outcome of what has been learned in their wards.

Heavy as have been our losses on the field of battle, and great as has been the crowd of wounded coming under the care of the medical department, the work of attending to them has been well done, and by the perfection to which antiseptic surgery has been carried, those who have had the misfortune to be wounded have been at least been spared those terrible outbreaks of septic disease which in former wars too often turned military hospitals into pest-houses.

All the modern improvements in the treatment of wounds which have had the effect of at once eliminating one of the most terrible aspects of war, have been the direct outcome of the work which has been done, and the investigations which have been made, during the past 30 years in our great charity-supported hospitals; and, so far from it being excusable for people to forget Hospital Sunday, or to say that their interests now are centred in other things, those whose minds are now full of war, and whose thoughts are concentrated upon the doings of their friends and relatives "at the front," ought now above all times to give a thankoffering to those great hospitals in whose wards during times of peace methods of surgical treatment have been evolved by aid of which many of the most terrible of the horrors of war have been wiped away. This is the thought which we offer for the consideration of those who would put aside the consideration of the peaceful work of hospitals to a more convenient season, as being something almost inappropriate to times like these. To such we would suggest, nay, we would assert, that, putting on one side the intrepid bravery of our troops as a thing always to be relied upon, the one bright spot in the conduct of the war has been the care, the skill, and, be it added, the success with which the sick and wounded have been cared for, all of which is the direct outcome of the work done, and the experience gained, in charity hospitals, and that these charity hospitals well deserve full recognition by a grateful people and full coffers on Hospital Sunday.

It is not, we think, out of place to recall on such an occasion as the present the fact that in all the older wars, and even in the last great war in which England has been involved, military hospitals have been constantly overrun with septic diseases of a most malignant type. Erysipelas used to break out whenever the wards were crowded, pyæmia was so continually present that wounds involving bones came to be regarded as almost fatal injuries, while that horrible disease which in the old wars earned for itself the name of hospital gangrene (so common was it in military hospitals in those days) used to sweep through the wounded like a ghastly epidemic, attacking slight wounds almost as often as severe ones, and rendering the hospitals at the seat of war almost as dangerous as the battle-fields. War always has its horrors. Nothing can alter that. What is new is that the hospitals are now able to do so much to mitigate them; and for all this we owe our thanks to the methods of treatment evolved in the wards of the charity hospitals.

This is not the place to tell the interesting, the fascinating story of Lord Lister's discovery of the relation between septic diseases and the growth of living micro-organisms in the wounds which they affected, or to show how, applying the discoveries which Pasteur had made in regard to fermentation, to explain what was going on in the human frame during the progress of these diseases, he was enabled to devise an antiseptic method by which these terrible scourges could be banished from the wards of our hospitals. Nor need we more than allude to the enormous amount of work done not by Lord Lister alone, but by a great array of hospital surgeons throughout the country, all striving to perfect the methods by which septic disease might be kept at bay. What we have to point out is that these efforts have been successful, and that all the work which has led to this great success has been done in charity-supported hospitals. It is then to these hospitals that we owe the fact that we now have at our disposal a power and a control over the progress of wounds produced in war such as our fathers never possessed, and that now in consequence of the good work done in our voluntary hospitals we find that our wounded soldiers recover, sometimes almost without a scar, from injuries which would inevitably and without a grain of doubt have been mortal in the days of the Crimea.

All over this land are people who have relatives are at the seat of war. In every family are brothers, husbands, fathers, relatives, and friends of one degree or another of relationship and of dearness, who are at the present moment exposed to danger and to risk of wound in battle. Every one of these, then, owes a debt of gratitude to our great voluntary hospitals for the opportunities they have afforded for this great research, and for those great improvements in surgical treatment which have resulted in giving back to so many of our wounded soldiers their health, their limbs, and even their lives.

Those who are able to look back on pre-antiseptic days and to compare the then and the now, and who, thinking of what was seen day after day in the hospitals at Scutari, contrast that with the records which now come to us of recovery from wounds which then would have been absolutely fatal, know, as others cannot know, that the soldiers and the friends of soldiers of to-day owe endless gratitude to our civil hospitals, even though they may have never been inside one, and that on Hospital Sunday the whole nation may properly be asked to contribute liberally to the support of our voluntary hospitals.

### THE ROLL CALL OF THE SICK.

IN order to enable our readers to readily realise the proportion borne to one another by the various classes of disease from which the inhabitants of London suffer, we have prepared the following set of diagrams, all drawn to the same scale, which show by their several sizes how the different classes of disease for which patients were treated during 1898 compare one with another in regard to the numbers of those affected by them.

The cases which we have sorted out in this way comprise those treated at the voluntary hospitals and dispensaries of London—including the endowed hospitals, St. Bartholomew's, Guy's, and St. Thomas's—and also at the hospitals of the Metropolitan Asylums Board. They amount altogether to the enormous total of *one million seven hundred and eighty-eight thousand five hundred and sixty-four patients.* That is to say, the equivalent of two and a-half cities containing a population equal to Manchester and Salford, or of three cities of the size of Liverpool, applied to the London hospitals for relief in the year 1898.

Dividing these into men, women, and children, we arrive at this result—that of the 1,788,564 patients, 679,308 were men, 595,261 women, and 513,995 children.

**Patients Suffering from Surgical Diseases.**—Of the whole number of patients received by the hospitals *eight hundred and eighteen thousand one hundred and sixty-six* required surgical treatment. What is meant by "surgical diseases?" They include all accidents, *i.e.*, broken bones, smashed limbs, fractured skulls, and all manner of fractures, displacements, and crushings of sensitive parts or organs. They further include abscesses, ulcerations, cancers, and tumours of all kinds; and, indeed, every injury which accident or pathological process may produce. Surgical diseases include all accidents and all lesions which may be dealt with by hand or instrument. Let anyone who desires to realise what surgical diseases mean for the population of London try to realise this army of 818,166 persons suffering from one or more of the injuries here briefly summarised.

**818,166.**
**Surgical Patients.**

**Patients Suffering from Medical Diseases.**—*Six hundred and sixteen thousand eight hundred and thirty-three* cases received medical treatment. What does the medical profession mean by medical diseases? Diseases which are situated in their entirety or as to their source and origin in one or other of the three great cavities of the body, many of them deep-seated, most of them removed from sight, the diagnosis of their nature and extent is dependent upon the scientific knowledge of the doctor to whose treatment they are committed. They include rheumatic fever, pneumonia, pleurisy, bronchitis, every kind of heart disease, many forms of brain lesion, diseases of the stomach, bowels, liver, kidney, bladder, pancreas and spleen, most nervous diseases, dyspepsia, constipation, headaches, sleeplessness, and a myriad other ailments, many of them serious, and often resulting in grave danger to life, or at least to the useful existence of mankind. Imagine for a moment considerably more than half a million human beings in London alone attended, free of cost to the patients, by the leading physicians of the day, within the buildings of the hospitals of London.

**616,833.**
**Medical Patients.**

**Patients Suffering from Eye Affections.**—*One hundred and twenty-two thousand and seventy-three* persons were treated in the special departments of the general hospitals or by the ophthalmic hospitals of London. It will be seen that, apart from the blind, there were nearly an eighth of a million people suffering from various forms of disease of the eye which often entailed excruciating pain, and must in many cases have terminated in loss of sight had it not been for the treatment they received at the hospitals. Those who are blessed with sight will surely give something to the hospitals on Hospital Sunday as a thank-offering for their escape from one of the most cruel diseases to which the human frame is liable.

**122,073.**
**Eye.**

**Patients Treated at Special Hospitals for Children.**—*One hundred and thirteen thousand three hundred and fifty-two* young people grievously ill sent from homes where they could not be properly treated or even carefully attended to. Every sympathetic person who studies what children have to suffer, how excellent is the skilled medical and surgical care which the hospitals supply to the many cases which arise amongst a large population like that of the metropolis of the British Empire must surely feel that no words should be needed to make the younger residents in this great city determine to give something, even from a limited income, to help suffering children to secure a return to health, and the power of one day shielding themselves against the dangers and risks to life of a vast city like London.

**113,352.**
**Children.**

### THE ROLL CALL OF THE SICK—*continued.*

77,025. Women.

**Diseases of Women and Motherhood.**—*Seventy-seven thousand and twenty-five* women were treated for the special diseases of women at the metropolitan voluntary hospitals, the major portion of whom attended the hospitals for women and the lying-in institutions. Sons of good mothers, and, indeed, all sons must necessarily be moved to pity by realising that, apart altogether from the diseases to which all are liable, woman has to face others which are peculiar to her sex, and which entail an immensity of suffering, if they do not render the sufferer permanently disabled from the enjoyment of life, and the pursuit of occupations which render life happy and profitable. This group of diseases must excite the sympathies of the most careless, and we make bold to believe that it will stir up many persons to give something this year to the hospitals if they have never yet enjoyed the luxury of investing a handsome sum in so noble a cause.

48,019. Ear and Throat.

**Patients Suffering from Diseases of the Ear and Throat.**—*Forty-eight thousand and nineteen* persons were treated at the special hospitals or special departments devoted to these diseases. The ear, throat, and nose are intimately connected, and large numbers of people require a visit to one of the special hospitals devoted to the ailments included in this section. Residents in a city like London, including both children and adults, are specially liable to these affections, which may involve temporary or permanent impairment of hearing, swallowing, breathing, and even speaking. Consider what it would mean to anyone of us to suffer from one or all of these affections. A little quiet reflection will surely awaken sympathy for those who thus suffer.

43,735. Skin.

**Patients Suffering from Diseases of the Skin.**—*Forty-three thousand seven hundred and thirty-five* patients were treated for skin diseases in London during the year. When it is remembered that many people of both sexes are terrified by the appearance of any affection of the skin, even in these days of advanced science, when immediate alleviation is possible, and permanent relief in the majority of cases assured, little need be said to awaken the sympathy of the reader for the many thousands who annually suffer from ailments of this description. We confidently claim a liberal offering from every inhabitant of London towards the funds needed to rescue those who have to suffer from the extremely common but essentially disagreeable illnesses included in this section.

31,573. Consumption.

**Patients Suffering from Consumption.**—*Thirty-one thousand five hundred and seventy-three* persons suffering from phthisis or consumption were treated at the consumption hospitals of London during the year. Consumption is the curse of our country. It spares neither the peer nor the pauper. It attacks the young, the old, and the middle-aged, whilst its ravages have often defied the highest skill of the greatest physicians. Who can find it in his heart to deny something towards the cost of alleviating the sufferings of those who have been stricken with this terrible malady, and of helping to prevent the spread of the disease to other members of the families of the sufferers?

16,721. Paralysis.

**Patients Suffering from Paralysis and Diseases of the Nerves.**—*Sixteen thousand seven hundred and twenty-one* people were stricken with paralysis and diseases of the nerves, and received treatment at the several hospitals devoted to these maladies. In the morning a man rises from his couch in robust health, and leaves his home to pursue his ordinary avocations. Whilst he is in the counting-house, or the study, or the office, or in the shop following his ordinary business, paralysis seizes him, and he is carried home insensible, helpless, and incapable of uttering a word. No disease is more appalling in its suddenness than paralysis, and it should be matter for thankfulness to Londoners that the hospitals and dispensaries of London were able during the year to give succour to no less than 16,721 cases of paralysis and diseases of the nerves. No one can doubt the association of nervous breakdown with the toil and moil of London life. Let those who are still spared give freely to relieve those who have fallen out of the race, and to keep open the hospitals where immediate treatment can be given to whomsoever may be struck down by those diseases.

14,419. Fever.

**Patients Suffering from Fever.**—The number of people who suffered from the various forms of fever during the year amounted to 14,419. Of this number over 12,000 were treated at the hospitals of the Metropolitan Asylums Board. But the term fever includes much besides the class of fevers which are usually removed to these hospitals. All over London measles prevails so largely as to cause three times as many deaths as occur from scarlet fever, and more even than take place from diphtheria, and all these innumerable cases of measles have to be attended in the homes of the people. Moreover, of the diseases notified in 1898 41·8 per cent. of the scarlet fever cases, 58·4 per cent. of the diphtheria cases, and 57·1 per cent. of the typhoid fever cases were not removed to the Asylums Board hospitals. Except the typhoid cases which were taken to the general hospitals, and some of the diphtheria cases which were carried off to these institutions in order that operations should be done to prevent impending suffocation by the disease, the great mass of these patients were treated in their own homes. The treatment of fever in its various forms has then fallen in a very much larger degree than many people imagine to the medical officers of the many charitable dispensaries scattered over London.

### A Word to Living Londoners.

### Ways and Means.

In our Special Hospital Sunday Supplements in former years we have given statistics to show the different proportions of the money given for the care of the sick in the metropolis by the living—namely, the present inhabitants of London, by deceased benefactors, and by the patients themselves; and we have shown that if the amount received from patients' payments be credited to the living, then the sum received by the hospitals from the living had only just about equalled that contributed by deceased benefactors. This, we have urged, is not an adequate proportion for the living to give; and, although there has been an improvement since these statistics were last given, there is much yet to be done. By the returns for the year 1898 we find that, including St. Bartholomew's Hospital, the living gave 9s. 5d. in the pound of the total income received by the London hospitals, which, added to the 1s. 2d. in the pound received from the patients' payments brings the contributions of the living up to 10s. 7d. in the pound in 1898. This result, judged by the returns of former years, is doubtless encouraging; but at the same time it does not remove from us the reproach that our great hospitals, of which London is so proud, are not supported either by the patients who throng their wards and waiting-rooms, or by the governors by whom they are managed, or even by the subscribers who look upon them, no doubt, as the outcome of their charity. They are really supported up to nearly one-half of their total receipts by the charity of those who have gone before, so that when we of the present generation look complacently upon the great hospitals around us as the proof of our Christian charity, we are taking to ourselves a credit which only very partially belongs to us. Surely it is our bounden duty at the present time to do at least something to remove this reproach, to increase our exertions on behalf of the medical charities of London, and to contribute liberally to the Hospital Sunday Fund.

### The Income Available for the Work Done.

In the year 1898 hospital treatment was provided for patients numbering about one million and three-quarters, exclusive of the 25,725 fever patients treated at the hospitals of the Metropolitan Asylums Board, and the total income which the London voluntary hospitals and dispensaries received during the year 1898 for this purpose was £1,054,823, which was derived from the following sources:—

| | |
|---|---|
| Charitable or voluntary contributions ... ... ... | £490,907, or 47 %. |
| Income from invested property ... ... ... | 257,146, or 24 %. |
| Legacies ... ... ... | 241,701, or 23 %. |
| Patients' payments ... ... | 65,069, or 6 %. |

So far as the above figures refer to St. Bartholomew's Hospital the income has been confined to that portion which was applicable to hospital purposes.

### How the Money is Provided.

It is of infinite importance that everyone should understand where the money comes from to pay the cost of the hospital relief given to the inhabitants of London by these voluntary institutions; and since we wish to bring the facts home to everybody, even to those to whom cut-and-dried statistics are sometimes meaningless and often unconvincing, we have had diagrams prepared, each representing a hand and a coin, which have been drawn to scale, and which on the one hand show the proportion of every sovereign received which was contributed by those of this generation which obtains all the benefits, and on the other hand the proportion contributed by deceased benefactors, many of whom, be it remembered, also took an active part in

The Dead Hand Gives 9s. 5d. Out of Every £1 received by the Hospitals.

the management of hospitals during their lifetime, as well as leaving benefactions which have enabled these institutions to meet the ever-increasing needs of the inhabitants of London. For the sake of clearness, and to ensure the diagrams being thoroughly understood, they have been drawn so as to represent the proportion given of every sovereign in 1898 by (*a*) the dead, (*b*) the living, and (*c*) the patients themselves. *The black hand* and the coin held by it represent the dead hand, *i.e.*, the contributions from those now dead; *the white hand* represents the charitable contributions of the living, and the smallest coin the amount derived from patients' payments.

Of every sovereign received 9s. 5d., or nearly equal to *one-half, is derived from legacies and the interest upon gifts* of deceased benefactors, which have been invested in approved securities; 9s. 5d. out of every sovereign received has been *given in charity by* the present inhabitants of London—that is, *the living for the benefit of whose generation the hospitals exist*; and 1s. 2d. out of every sovereign received has been *contributed by the patients treated in hospitals*. It will be seen, therefore, that in the year 1898 the living, including patients' payments, gave rather more than the deceased benefactors, but, encouraging as this may be, the living cannot afford to relax their efforts on behalf of the hospitals in any way. Let us consider the figures carefully for a moment. In the first place it must be remembered a considerable portion of the 9s. 5d. in the pound is due to

The Living, *i.e.*, the Present Inhabitants gave 9s. 5d. of each £1 received by the Hospitals.

legacies secured during 1898, and this source of income must always be fluctuating and unreliable. It is true that during the years 1897, 1898, and 1899 the amount received in legacies increased each year; but we have only to turn back to 1896

**Patients' Payments yield**

Patients' Payments Supply 1s. 2d. of Each £1 Received by the Voluntary Hospitals.

to find an instance of a sudden drop when the income received from legacies fell about £40,000 from the previous year; and it will be readily understood, therefore, that a falling off in the income received from legacies in any year might bring us face to face with the necessity of making up a deficiency from other sources, and it is only by the contributions of the living being steadily increased that we can hope to avoid such a contingency. To achieve a fairly good result for one year is not enough. The effort must be a sustained one. Generally speaking, the year 1898 was a good one for the hospitals, but we fear this has not been maintained in 1899, so far at least as the amount given in charity is concerned; for, although the figures are not available from which to form a complete analysis, it is apparent that there is a falling off in the amount received from the living as compared to the amount received from them in the year under discussion; and, having regard to the ever-growing demand upon the hospitals, and the rapid extension of the population of London, we must urge a sustained effort on the part of the inhabitants of the metropolis on behalf of those institutions which care for the sick poor.

The Meaning of the Diagrams.

We would recommend the attention of all classes in London to the foregoing figures, and to a careful consideration of the facts they bring out. Were it not for the contributions of the dead hand a large number of the hospitals would have to be closed for want of funds. They show, moreover, that whereas the people of London resort to the voluntary hospitals in larger numbers than the population of any other city in the United Kingdom, they nevertheless do not adequately show their sense of the benefits they receive from these institutions; and whereas in provincial cities all classes of the population combine to provide the necessary funds in due proportion, in London the living, while demanding greater value in relief every year at the hands of the hospitals, are satisfied with paying for the benefits they receive a little over one-half of this total cost, and are content to trust to the dead hand to supply the deficiency. When we consider what the position would be were the dead hand to fail us, even to a small extent, when we remember that there are hospitals which are urgently in need of large sums to defray their current expenses, and districts with teeming populations without adequate hospital accommodation at all, we must surely determine to emulate the charity of our ancestors, and to increase our contributions on Hospital Sunday, in order to remove from this great city the reproach of living on the charity of the dead, and to render any check in the great work of our hospitals an absolute impossibility.

### The Claims of Convalescent Homes.

The immense value of Convalescent Homes has been demonstrated in a striking manner since our wounded and invalided soldiers commenced to return from the front. Though a certain proportion of the men are patients needing surgical operations or medical treatment, by far the great majority leave South Africa almost recovered from injury or illness, and on their arrival in this country they chiefly require plenty of fresh air, good food, and rest. In fact, as Miss Norman has testified, Netley and some of the other military institutions at this time more closely resemble convalescent homes than ordinary hospitals. Yet one has only to stroll through the wards or grounds at Netley or Woolwich, and talk to the convalescents themselves, to learn what a serious matter it would have been for them if they had been compelled to go back at once to their homes, or to attempt to engage in any kind of labour. They want little nursing or medicine, but they want a great deal of building up. In other words, their prospects of being completely restored to health and strength and able to earn their living again, would be small indeed if they were not kept for some weeks in the convalescent homes of the Army.

Our Duty to Civilians.

Happily, what the Army does for our soldiers, private individuals endeavour to do for our civilians, and the only reason why more is not accomplished is lack of the indispensable means. This is the time to remind the British public that the claims of convalescent homes upon their support are not less binding than those of hospitals, a fact which is fully recognised by the Council of the Metropolitan Hospital Sunday Fund in their allocation of sums to the charities. Many of the homes are, indeed, attached to London hospitals, and carried on under the auspices of the authorities.

### A Pathetic Sight.

There is scarcely a more pathetic sight in the world than that of a patient discharged as "cured" from one of the great general hospitals, who is still obviously not only too weak to work, but is also quite unfit to face the privations of existence in a crowded slum. But a great general hospital cannot have its beds occupied by persons who have ceased to demand the attention of the medical and nursing staff; and unless they can obtain admission to a convalescent home, they must take their chance of struggling back to health under the most depressing conditions. Even when it is the possessor of a well-ordered home who has been ill he knows that the stage of convalescence, when the doctor has given up calling and the nurse has gone away, is hard to bear, and that it is not until there has been the longed-for change of air and scene that the effect of protracted pain and confinement begins to wear off. How infinitely worse must it be for those whose change after a protracted illness is from the spacious, pleasant ward of a hospital, with a comfortable bed and ample food, to a dingy attic in a close court, with scarcely bread enough to eat? Is it at all surprising that many who recover from an injury or disease in hospital fade and die in the stage of convalescence, because no man careth for their bodies? This is why we so earnestly commend the claims of convalescent homes that have been established to finish the splendid work, which the hospitals have begun, to the utmost extent of their powers. The charity that stops short of setting the man upon his legs again, of enabling the woman to resume her household duties, of bringing back the roses to the cheeks of children, is not sufficient. It is our duty first as Christians, and then as citizens, to see to it that when patients are discharged from hospital they are not thrown upon their own resources, to become chronic invalids or to endure, as convalescents, a form of suffering to which they were strangers as patients.

### A Hopeful Sign.

The multiplication of convalescent homes is one of the most hopeful signs of the times. It shows, at all events, that the lesson of duty to our neighbour is gradually being better learnt; that the Good Samaritan is, by slow degrees, winning the world to His methods of mercy. To-day there are many exampes of a kind of institution which not so very long ago was absolutely non-existent. The founders of convalescent homes have advantages which are denied to the founders of hospitals. They can build on sites which are comparatively cheap, and there is no difficulty in securing ample space. Of course, the position must be known to be healthy, and a glance at the list of homes leaves no room for doubt that this condition has been fulfilled. If the accommodation is meagre and the grounds are circumscribed, it is merely because the funds have not justified further expenditure. We have heard this year of several cases of impoverishment, involving the refusal of admission to many in sore need. It will always be so, perhaps; but at any rate, we should not be open as a nation to the reproach that while we are willing to contribute freely, if not lavishly, for the benefit of sick and wounded soldiers, we withdraw our mite which we have hitherto given to charities equally deserving. The convalescent homes, whether they are for men, or women, or children; for the victims of accidents or of scarlet fever; whether they are situated on bracing heights near London, or within reach of the delicious sea breeze; whether they wholly depend upon the support of the charitable, or whether they only require supplemental assistance, must be regarded as part and parcel of the great hospital movement which attests the reality of our religion in the eyes of multitudes whom the voice from the pulpit does not reach.

### A General Appeal.

Here we make no appeal for a particular institution. Our plea is that the pressing needs of the convalescent homes supply one of manifold reasons why the Hospital Sunday Fund of 1900 should be larger than on any past occasion. They will get their share according to the amount which is collected in the churches and chapels on Sunday, or sent in by cheque to the Mansion House. It is true that their expenses of management are usually extremely moderate. If physicians or surgeons are wanted their services are freely accorded, and honorary secretaries are the rule. There is generally a ladies' visiting committee in connection with the home, and the members devote themselves ungrudgingly to the work. But these things should be an incentive to giving, for they mean that the bulk of the money subscribed is spent in the interest of the convalescents, and that the most admirable precautions are taken to guard against waste. It is not a mere Utopian dream that every hospital should have its convalescent home. Meanwhile, the call to maintain and enhance the usefulness of those which already provide the pleasant shelter, the best of food, and the entire freedom from anxiety, which the convalescent poor need no less than the convalescent well-to-do, sounds in the ears of every man and woman who understands the real purpose of life, above the confusing din of party politics, the frivolous chatter of society, and the imperious demands of personal aggrandisement.

And thus we come back to Hospital Sunday, and to the urgent necessity which lies upon us to increase the Fund, so that a far larger amount shall be available with which to help the convalescent institutions than it has hitherto been possible to devote to such a purpose. So far the help given by the Fund has been chiefly of an indirect nature, through the hospitals which are equipped with convalescent branches. But a beginning has been made in the giving of direct help to these deserving institutions, and nothing but lack of money prevents that help being greater still, and far more effectual than at present in relieving one of the greatest wants of the sick poor.

### Metropolitan Hospital Sunday Fund, 1900.

### A YEAR'S WORK IN THE HOSPITALS AND MEDICAL CHARITIES OF LONDON.

### NEWINGTON AND SOUTH DISTRICT.

Comprising Battersea, Wandsworth, Tooting, Balham, Streatham, Brixton, Lambeth, Newington, Southwark, Bermondsey, Camberwell, Greenwich, Deptford, Lewisham, Blackheath, Woolwich, &c.

| No. of Beds. | No. of Beds Daily Occupied. | HOSPITALS. | In-patnts. | Out-patients. | Total Expenditure. | Income. Charitable. | Proprietary. | Patnts' pymnts. | Total Income. | Legacies not included in previous column. |
|---|---|---|---|---|---|---|---|---|---|---|
| | | | | | £ | £ | £ | £ | £ | £ |
| 650 | 458 | Guy's ... ... ... ... ... ... ... | 7,120 | 98,860 | 80,935 | 14,205 | 30,645 | 3,984 | 48,834 | 24,890 |
| 10 | 5 | Phillips' Memorial Homœopathic ... ... | 10 | 5 | 554 | 345 | 57 | 200 | 602 | 100 |
| 25 | 20 | Miller ... ... ... ... ... ... ... | 252 | 17,918 | 3,546 | 2,675 | 352 | ... | 3,027 | 100 |
| 248 | 212 | Seamen's... ... ... ... ... ... ... | 2,515 | 23,282 | 19,033 | 7,990 | 4,238 | 529 | 12,757 | 4,645 |
| 570 | 400 | St. Thomas's ... ... ... ... ... ... | 5,831 | 65,940 | 59,406 | 5,324 | 50,056 | 346 | 55,726 | 13,443 |
| 66 | 56 | Evelina, for Children ... ... ... ... | 1,063 | 19,489 | 6,001 | 2,458 | 3,593 | 100 | 6,151 | 20,000 |
| 57 | 48 | Home for Sick Children ... ... ... | 229 | 1,432 | 1,818 | 1,103 | 215 | 346 | 1,664 | 270 |
| 24 | 22 | General Lying-in ... ... ... ... ... | 560 | 1,667 | 3,409 | 698 | 2,665 | ... | 3,363 | ... |
| 18 | 15 | Clapham Maternity and Dispensary... ... | 335 | 5,816 | 2,321 | 680 | 1,055 | 892 | 2,627 | ... |
| 54 | 50 | Royal, for Children and Women ... ... | 627 | 9,334 | 4,472 | 1,849 | 962 | 258 | 3,069 | 583 |
| 42 | 35 | Royal Eye ... ... ... ... ... ... | 570 | 17,929 | 3,527 | 5,573 | 158 | 664 | 6,395 | ... |
| ... | ... | Hospital for Diseases of the Skin ... ... | ... | 5,397 | 976 | 188 | 236 | 600 | 1,024 | ... |
| 11 | 8 | Eltham Cottage... ... ... ... ... ... | 130 | ... | 513 | 454 | ... | 141 | 595 | ... |
| 30 | 7 | Beckenham Cottage ... ... ... ... ... | 111 | ... | 605 | 597 | 14 | 67 | 588 | 23 |
| 18 | 12 | Blackheath Cottage ... ... ... ... ... | 190 | 1,052 | 952 | 720 | 24 | 110 | 854 | ... |
| 34 | 25 | Bromley Cottage ... ... ... ... ... | 356 | ... | 1,389 | 1,006 | 187 | 192 | 1,385 | 4,710 |
| 14 | 10 | Chislehurst, &c., Cottage ... ... ... .. | 160 | ... | 660 | 429 | 11 | 177 | 617 | ... |
| 12 | 6 | Sidcup Cottage ... ... ... ... ... | 138 | 103 | 529 | 402 | 10 | 112 | 524 | ... |
| 7 | 5 | Shortlands Convalescent ... ... ... ... | 97 | ... | 179 | 169 | 1 | 5 | 175 | ... |
| 16 | 12 | Livingstone Cottage ... ... ... ... | 145 | ... | 743 | 748 | 8 | 53 | 809 | 500 |
| 1,906 | 1,406 | | 20,439 | 268,224 | 191,568 | 47,523 | 94,487 | 8,776 | 150,786 | 69,264 |
| | | DISPENSARIES. | | | | | | | | |
| ... | ... | Battersea Provident ... ... ... ... ... | ... | 29,846 | 3,207 | 109 | 61 | 3,101 | 3,271 | ... |
| ... | ... | Brixton, &c. ... ... ... ... ... ... | ... | 4,169 | 650 | 511 | 37 | 77 | 625 | ... |
| ... | ... | Camberwell Provident ... ... ... ... | ... | 9,364 | 1,902 | 387 | 137 | 1,469 | 1,993 | ... |
| ... | ... | Clapham ... ... ... ... ... ... ... | ... | 1,463 | 406 | 227 | 15 | 126 | 368 | 50 |
| ... | ... | Deptford Medical Mission ... ... ... ... | ... | 2,580 | 374 | 256 | 31 | 84 | 371 | ... |
| ... | ... | East Dulwich Provident ... ... ... ... | ... | 4,533 | 800 | 101 | 2 | 731 | 834 | ... |
| ... | ... | Forest Hill ... ... ... ... ... ... | ... | 2,323 | 712 | 262 | 22 | 469 | 753 | ... |
| ... | ... | Greenwich Provident ... ... ... ... ... | ... | 4,162 | 611 | 43 | 15 | 544 | 602 | ... |
| ... | ... | Royal South London ... ... ... ... ... | ... | 4,773 | 624 | 525 | 114 | ... | 639 | ... |
| ... | ... | South Lambeth, &c. ... ... ... ... ... | ... | 2,430 | 562 | 354 | 19 | 194 | 567 | ... |
| ... | ... | Walworth Provident ... ... ... ... ... | ... | 760 | 288 | 50 | 28 | 151 | 229 | ... |
| ... | ... | Wandsworth Common... ... ... ... ... | ... | 1,002 | 226 | 50 | ... | 175 | 225 | ... |
| ... | ... | Woolwich, &c., Provident ... ... ... ... | ... | 1,063 | 364 | 51 | 11 | 252 | 314 | ... |
| 1,906 | 1,406 | | 20,439 | 336,692 | 202,294 | 50,449 | 94,979 | 16,149 | 161,577 | 69,314 |

### CITY AND EAST CENTRAL DISTRICT.

Comprising the City, St. Luke's, Shoreditch, Finsbury, and Clerkenwell.

| No. of Beds. | No. of Beds Daily Occupied. | HOSPITALS. | In-patnts. | Out-patients. | Total Expenditure. | Income. Charitable. | Proprietary. | Patnts' pymnts. | Total Income. | Legacies not included in previous column. |
|---|---|---|---|---|---|---|---|---|---|---|
| | | | | | £ | £ | £ | £ | £ | £ |
| 72 | 70 | Metropolitan ... ... ... ... ... ... | 867 | 31,245 | 9,660 | 6,372 | 448 | 441 | 7,261 | 2,704 |
| 170 | 127 | Royal Free ... ... ... ... ... ... | 1,984 | 33,167 | 11,838 | 4,928 | 1,150 | ... | 6,078 | 5,853 |
| 80 | 62 | Royal, for Diseases of the Chest ... ... | 798 | 6,671 | 7,493 | 6,586 | 144 | ... | 6,730 | 1,633 |
| 57 | 47 | North-Eastern, for Children ... ... ... | 748 | 16,665 | 5,828 | 4,950 | 489 | 670 | 6,109 | 100 |
| 36 | 26 | City of London Lying-In ... ... ... | 585 | 1,559 | 5,709 | 492 | 3,625 | 3 | 4,120 | 100 |
| 45 | 29 | St. Mark's, for Fistula ... ... ... | 362 | 1,016 | 4,019 | 2,372 | 673 | ... | 3,045 | 1,754 |
| 138 | 64 | Royal London Ophthalmic ... ... ... | 1,919 | 37,832 | 8,890 | 3,750 | 648 | ... | 4,398 | 509 |
| 60 | 21 | City Orthopædic ... ... ... ... | 131 | 2,422 | 3,295 | 2,520 | 113 | ... | 2,633 | ... |
| 17 | 10 | Central London Throat and Ear ... ... | 272 | 8,843 | 2,403 | 594 | 88 | 1,792 | 2,474 | ... |
| 675 | 456 | | 7,666 | 139,420 | 59,135 | 32,564 | 7,378 | 2,906 | 42,848 | 12,653 |
| | | DISPENSARIES. | | | | | | | | |
| ... | ... | City ... ... ... ... ... ... ... | ... | 4,492 | 1,318 | 888 | 233 | ... | 1,121 | 425 |
| ... | ... | City of London and East London ... ... | ... | 23,644 | 1,680 | 105 | 70 | 1,913 | 2,088 | 25 |
| ... | ... | Farringdon General ... ... ... ... | ... | 3,167 | 792 | 670 | ... | 202 | 872 | ... |
| ... | ... | Finsbury... ... ... ... ... ... ... | ... | 14,247 | 1,094 | 369 | 164 | 269 | 802 | 578 |
| ... | ... | Metropolitan ... ... ... ... ... ... | ... | 5,870 | 751 | 385 | 126 | 257 | 768 | 90 |
| ... | ... | Royal General ... ... ... ... ... ... | ... | 3,354 | 773 | 327 | 456 | 95 | 878 | 50 |
| 675 | 456 | | 7,666 | 194,194 | 65,543 | 35,308 | 8,427 | 5,642 | 49,377 | 13,821 |

### ST. MARYLEBONE AND WEST CENTRAL DISTRICT.

Comprising St. Marylebone, St. John's Wood, Bloomsbury, Holborn, &c.

| No. of Beds. | No. of Beds Daily Occupied. | HOSPITALS. | In-patnts. | Out-patients. | Total Expenditure. | Income. Charitable. | Income. Proprietary | Income. Patnts' pymnts. | Total Income. | Legacies not included in previous column. |
|---|---|---|---|---|---|---|---|---|---|---|
| | | | | | £ | £ | £ | £ | £ | £ |
| 70 | 49 | French ... ... ... ... ... ... ... | 871 | 4,704 | 3,864 | 4,154 | 8 | ... | 4,162 | 2,144 |
| Rebui | lding. | Italian ... ... ... ... ... ... ... | ... | 4,943 | 510 | 1,303 | 210 | ... | 1,513 | ... |
| 103 | 80 | London Homœopathic ... ... ... ... | 1,128 | 20,678 | 10,396 | 10,243 | 3,254 | 911 | 14,408 | 2,848 |
| 24 | 9 | SS. John and Elizabeth ... ... ... | 20 | ... | 1,890 | 966 | 807 | ... | 1,773 | 397 |
| 372 | 336 | The Middlesex ... ... ... ... ... | 4,427 | 49,817 | 33,515 | 13,798 | 8,835 | ... | 22,633 | 9,834 |
| 68 | 22 | Alexandra for Children ... ... ... | 65 | 350 | 2,194 | 3,443 | 111 | 123 | 3,677 | ... |
| 30 | 26 | Hospital for Incurable Children ... ... | 34 | ... | 1,262 | 431 | 86 | 335 | 852 | 1,050 |
| 238 | 170 | Hospital for Sick Children ... ... ... | 2,175 | 23,892 | 19,418 | 7,633 | 3,844 | ... | 11,477 | 2,597 |
| 20 | 14 | British Lying-in ... ... ... ... | 331 | 419 | 2,162 | 510 | 1,932 | 90 | 2,532 | 300 |
| 63 | 51 | Queen Charlotte's Lying-in ... ... ... | 1,254 | 1,011 | 4,730 | 3,588 | 288 | 120 | 3,996 | 2,350 |
| 50 | 42 | New Hospital for Women ... ... ... | 594 | 14,485 | 5,202 | 2,922 | 205 | 1,539 | 4,666 | 100 |
| 50 | 41 | Samaritan Free... ... ... ... ... | 517 | 7,430 | 6,158 | 4,606 | 217 | ... | 4,823 | 550 |
| 200 | 176 | National for the Paralysed, &c. ... ... | 1,032 | 6,170 | 15,079 | 5,566 | 1,750 | 2,579 | 9,895 | 4,168 |
| 25 | 16 | Hospital for Epilepsy, &c. ... ... | 90 | 769 | 1,634 | 812 | 29 | 507 | 1,348 | 3,170 |
| 50 | 41 | West End, for Epilepsy, &c.... ... ... | 325 | 3,480 | 3,241 | 3,072 | 91 | 620 | 3,783 | ... |
| 28 | 18 | Central London Ophthalmic ... ... ... | 402 | 11,925 | 1,622 | 1,607 | 29 | ... | 1,636 | ... |
| 13 | 6 | Western Ophthalmic ... ... ... ... | 138 | 8,290 | 950 | 718 | 139 | 4 | 861 | ... |
| 60 | 50 | National Orthopædic ... ... ... ... | 203 | 765 | 2,283 | 1,218 | 9 | 886 | 2,113 | 60 |
| 20 | 13 | Establishment for Gentlewomen ... ... | 138 | ... | 2,530 | 879 | 111 | 895 | 1,885 | 1,185 |
| ... | ... | National Dental ... ... ... ... | ... | 26,641 | 1,367 | 498 | ... | 1,063 | 1,561 | ... |
| 16 | 8 | London Throat... ... ... ... ... | 579 | 4,757 | 1,411 | 430 | ... | 1,032 | 1,462 | ... |
| 8 | 1 | Metropolitan Ear, Nose, and Throat ... | 103 | 3,087 | 447 | 255 | ... | 189 | 444 | ... |
| 1,508 | 1,169 | DISPENSARIES. | 14,426 | 193,613 | 121,865 | 68,652 | 21,955 | 10,893 | 101,500 | 30,753 |
| ... | ... | Bloomsbury Provident ... ... ... | ... | 1,012 | 282 | 33 | ... | 213 | 246 | ... |
| ... | ... | London Medical Mission ... ... ... | ... | 8,328 | 1,356 | 843 | 55 | 270 | 1,168 | 450 |
| ... | ... | Infirmary for Consumption ... ... ... | ... | 493 | 637 | 175 | 187 | ... | 362 | ... |
| ... | ... | Portland Town... ... ... ... ... | ... | 1,352 | 151 | 108 | 4 | 10 | 122 | ... |
| ... | ... | St. John's Wood Provident ... ... ... | ... | 5,641 | 633 | 256 | 72 | 413 | 741 | ... |
| ... | ... | St. Marylebone General ... ... ... | ... | 3,086 | 776 | 418 | 161 | 195 | 774 | 250 |
| ... | ... | Western General ... ... ... ... | ... | 17,263 | 1,310 | 1,068 | 27 | 61 | 1,156 | 11 |
| 1,508 | 1,169 | | 14,426 | 230,788 | 127,010 | 71,553 | 22,461 | 12,055 | 106,069 | 31,464 |

### KENSINGTON AND WEST DISTRICT.

Comprising Kensington, Paddington, Bayswater, Kilburn, Chelsea, Brompton, Fulham, Hammersmith, Chiswick, Brentford, Acton, Ealing, &c.

| No. of Beds. | No. of Beds Daily Occupied. | HOSPITALS. | In-patnts. | Out-patients. | Total Expenditure. | Income. Charitable. | Income. Proprietary | Income. Patnts' pymnts. | Total Income. | Legacies not included in previous column. |
|---|---|---|---|---|---|---|---|---|---|---|
| | | | | | £ | £ | £ | £ | £ | £ |
| 351 | 323 | St. George's ... ... ... ... ... ... | 4,276 | 29,713 | 44,868 | 9,691 | 15,501 | 99 | 25,291 | 4,402 |
| 281 | 198 | St. Mary's ... ... ... ... ... ... | 3,142 | 39,663 | 29,334 | 10,164 | 1,602 | ... | 11,766 | 17,247 |
| 153 | 124 | West London ... ... ... ... ... | 1,956 | 25,191 | 8,890 | 6,568 | 276 | ... | 6,844 | 310 |
| 321 | 274 | Hospital for Consumption ... ... ... | 1,514 | 7,486 | 34,769 | 13,734 | 7,822 | ... | 21,556 | 13,100 |
| 16 | 12 | Belgrave, for Children ... ... ... | 281 | 5,103 | 1,438 | 1,687 | 47 | ... | 1,734 | ... |
| 50 | 49 | Cheyne, for Sick and Incurable Children ... | 24 | ... | 2,735 | 1,709 | 367 | 330 | 2,406 | 1,152 |
| 46 | 38 | Paddington Green, for Children ... ... | 633 | 14,028 | 3,862 | 2,762 | 209 | 304 | 3,275 | 562 |
| 102 | 74 | Victoria, for Children ... ... ... ... | 730 | 17,022 | 6,800 | 4,200 | 656 | 332 | 5,188 | 1,250 |
| 52 | 38 | Chelsea, for Women ... ... ... ... | 587 | 2,438 | 7,458 | 5,166 | 235 | 781 | 6,182 | 879 |
| 105 | 86 | Cancer ... ... ... ... ... ... | 757 | 1,037 | 12,016 | 5,180 | 3,404 | ... | 8,584 | 13,741 |
| 135 | 64 | Female Lock ... ... ... ... ... | 534 | ... | 4,549 | 1,938 | 27 | 1,342 | 3,307 | 292 |
| 16 | 9 | Epsom and Ewell Cottage ... ... ... | 115 | ... | 995 | 646 | 10 | 172 | 828 | ... |
| 25 | 19 | Reigate and Redhill Cottage ... ... ... | 251 | ... | 1,286 | 600 | 49 | 150 | 799 | ... |
| 16 | 10 | Wimbledon Cottage ... ... ... ... | 146 | ... | 723 | 599 | 5 | 170 | 774 | 200 |
| 15 | 8 | Hounslow Cottage ... ... ... ... | 285 | ... | 597 | 322 | 195 | 55 | 572 | ... |
| 1,684 | 1,326 | | 15,231 | 141,681 | 160,320 | 64,966 | 30,405 | 3,735 | 99,106 | 53,135 |
| | | DISPENSARIES. | | | | | | | | |
| ... | ... | Brompton Provident ... ... ... ... | ... | 946 | 425 | 128 | 76 | 220 | 424 | ... |
| ... | ... | Chelsea, &c. ... ... ... ... ... | ... | 4,704 | 678 | 419 | 175 | ... | 594 | ... |
| ... | ... | Chelsea Provident ... ... ... ... | ... | 451 | 228 | 34 | ... | 150 | 184 | ... |
| ... | ... | Kensal Town Provident ... ... ... | ... | 701 | 301 | 25 | ... | 270 | 295 | ... |
| ... | ... | Kensington ... ... ... ... ... | ... | 3,255 | 565 | 524 | 49 | ... | 573 | 100 |
| ... | ... | Kilburn, Maida Vale ... ... ... ... | ... | 2,363 | 433 | 382 | 47 | ... | 429 | ... |
| ... | ... | Kilburn Provident ... ... ... ... | ... | 5,094 | 1,189 | 70 | 13 | 1,105 | 1,188 | ... |
| ... | ... | Notting Hill Provident ... ... ... | ... | 376 | 202 | 63 | 13 | 92 | 168 | ... |
| ... | ... | Paddington Provident... ... ... ... | ... | 3,131 | 550 | 153 | 23 | 355 | 531 | ... |
| ... | ... | Pimlico Provident ... ... ... ... | ... | 2,500 | 826 | 19 | ... | 872 | 891 | ... |
| ... | ... | Royal Pimlico Provident ... ... ... | ... | 3,600 | 864 | 386 | 13 | 576 | 975 | ... |
| ... | ... | Westbourne Provident ... ... ... ... | ... | 1,031 | 414 | 54 | 47 | 335 | 436 | ... |
| 1,684 | 1,326 | | 15,231 | 169,833 | 166,995 | 67,223 | 30,861 | 7,710 | 105,794 | 53,235 |

### ISLINGTON AND NORTH-WEST DISTRICT.

Comprising Islington, Holloway, Highbury, Hampstead, Highgate, St. Pancras, Stoke Newington, Tottenham, &c.

| No. of Beds. | No. of Beds Daily occupied | Hospitals. | In-patnts. | Out-patients. | Total Expenditure. | Income. Charitable. | Proprietary. | Patnts pymnts. | Total Income. | Legacies not included in previous column. |
|---|---|---|---|---|---|---|---|---|---|---|
| | | | | | £ | £ | £ | £ | £ | £ |
| 146 | 130 | Great Northern Central ... ... ... | 1,923 | 31,189 | 12,516 | 6,301 | 1,121 | 535 | 7,957 | 7,000 |
| 31 | 21 | Hampstead Hospital ... ... ... ... | 356 | 1,080 | 3,169 | 1,385 | 470 | 335 | 2,190 | ... |
| 120 | 80 | London Temperance ... ... ... ... | 1,354 | 20,873 | 10,703 | 7,943 | 1,026 | 340 | 9,309 | 2,747 |
| 53 | 47 | North-West London ... ... ... ... | 585 | 18,473 | 4,883 | 2,452 | 366 | 33 | 2,851 | 1,000 |
| 103 | 29 | Tottenham Training ... ... ... ... | 530 | 9,157 | 3,142 | 4,752 | 16 | 217 | 4,985 | 329 |
| 185 | 161 | University College ... ... ... ... | 2,708 | 37,562 | 17,356 | 8,895 | 3,442 | 30 | 12,367 | 13,494 |
| 80 | 72 | North London Consumption ... ... ... | 511 | 3,598 | 6,585 | 5,574 | 157 | 10 | 5,741 | 1,425 |
| 150 | 75 | London Fever ... ... ... ... ... | 629 | ... | 10,924 | 7,400 | 1,822 | 2,051 | 11,273 | 2,910 |
| 28 | 19 | Invalid Asylum ... ... ... ... ... | 195 | ... | 873 | 464 | 136 | 240 | 840 | ... |
| 15 | 12 | Children's Home Hospital, Barnet ... ... | 51 | ... | 549 | 414 | 61 | 70 | 545 | ... |
| 14 | 12 | Enfield Cottage ... ... ... ... ... | 172 | ... | 801 | 546 | 48 | 118 | 712 | 100 |
| 30 | 25 | Memorial Cottage, Mildmay ... ... ... | 196 | ... | 1,588 | 404 | 975 | 91 | 1,470 | ... |
| 28 | 14 | St. Saviour's Home ... ... ... ... | 119 | ... | 1,939 | 1,077 | 9 | 705 | 1,791 | ... |
| 42 | 32 | Friedenheim Home ... ... ... ... | 114 | ... | 3,555 | 2,156 | 65 | 169 | 2,390 | 500 |
| 34 | 26 | St. Monica's, Brondesbury ... ... ... | 47 | ... | 1,557 | 808 | 180 | 320 | 1,308 | 66 |
| 25 | 9 | Willesden Cottage ... ... ... ... | 142 | ... | 888 | 584 | 158 | 56 | 798 | ... |
| 1,084 | 764 | DISPENSARIES. | 9,632 | 121,932 | 81,028 | 51,155 | 10,032 | 5,320 | 66,527 | 29,571 |
| ... | ... | Camden Provident ... ... ... ... | ... | 1,022 | 316 | 19 | ... | 232 | 251 | ... |
| ... | ... | Child's Hill Provident ... ... ... ... | ... | 441 | 333 | 38 | 17 | 278 | 333 | ... |
| ... | ... | Hampstead Provident ... ... ... ... | ... | 11,074 | 1,012 | 222 | 32 | 758 | 1,012 | ... |
| ... | ... | Holloway and North Islington ... ... | ... | 3,765 | 769 | 301 | 45 | 328 | 674 | ... |
| ... | ... | Islington ... ... ... ... ... ... | ... | 12,402 | 874 | 284 | 19 | 556 | 859 | ... |
| ... | ... | St. Pancras and Northern ... ... ... | ... | 1,411 | 549 | 255 | 138 | 98 | 491 | ... |
| ... | ... | Stamford Hill, &c. ... ... ... ... | ... | 5,940 | 745 | 563 | 164 | ... | 727 | 25 |
| 1,084 | 764 | | 9,632 | 157,987 | 85,626 | 52,837 | 10,467 | 7,570 | 70,874 | 29,596 |

### WESTMINSTER DISTRICT.

Comprising Westminster City and Liberties.

| No. of Beds. | No. of Beds Daily occupied | Hospitals. | In-patnts. | Out-patients. | Total Expenditure. | Charitable. | Proprietary. | Patnts pymnts. | Total Income. | Legacies not included in previous column. |
|---|---|---|---|---|---|---|---|---|---|---|
| 175 | 151 | Charing Cross ... ... ... ... ... | 2,326 | 22,527 | 19,149 | 27,305 | 1,663 | 193 | 29,161 | 7,865 |
| 217 | 168 | King's College ... ... ... ... ... | 2,217 | 17,130 | 19,569 | 11,517 | 2,407 | 126 | 14,050 | 4,567 |
| 202 | 126 | Westminster ... ... ... ... ... | 1,866 | 22,785 | 20,700 | 5,910 | 2,941 | ... | 8,851 | 17,193 |
| 153 | 129 | Ventnor, for Consumption ... ... ... | 729 | ... | 21,955 | 5,297 | 2,221 | 3,583 | 11,101 | 10,446 |
| 22 | 17 | Grosvenor, for Women and Children ... | 189 | 3,032 | 2,306 | 1,263 | 12 | 525 | 1,800 | ... |
| 64 | 48 | Hospital for Women ... ... ... ... | 706 | 4,505 | 6,153 | 3,057 | 320 | 540 | 3,917 | 1,943 |
| 25 | 20 | National, for Diseases of Heart ... ... | 179 | 2,249 | 2,448 | 1,481 | 66 | 447 | 1,994 | 250 |
| 36 | 26 | Royal Westminster Ophthalmic ... ... | 610 | 10,449 | 2,331 | 1,746 | 609 | 56 | 2,411 | 2,102 |
| 50 | 24 | Royal Orthopædic ... ... ... ... | 126 | 671 | 3,812 | 1,233 | 544 | 85 | 1,862 | 400 |
| 9 | 8 | Royal Ear ... ... ... ... ... | 269 | 2,148 | 846 | 227 | ... | 579 | 806 | ... |
| ... | ... | Dental ... ... ... ... ... ... | ... | 35,584 | 2,006 | 2,469 | 241 | 178 | 2,888 | 100 |
| 27 | 11 | Gordon, for Fistula ... ... ... ... | 185 | 940 | 1,271 | 378 | ... | 904 | 1,282 | ... |
| 27 | 21 | St. Peter's, for Stone ... ... ... ... | 562 | 4,435 | 4,540 | 1,058 | 356 | 1,984 | 3,398 | ... |
| 50 | 32 | St. John's, for Skin ... ... ... ... | 281 | 7,434 | 3,956 | 1,557 | 13 | 2,451 | 4,021 | 50 |
| 33 | 29 | Oxygen Home ... ... ... ... ... | 72 | ... | 1,273 | 354 | 31 | 814 | 1,199 | ... |
| 1,090 | 810 | DISPENSARIES. | 10,317 | 133,889 | 112,315 | 64,852 | 11,424 | 12,465 | 88,741 | 44,916 |
| ... | ... | Public ... ... ... ... ... ... | ... | 2,962 | 722 | 385 | 194 | ... | 579 | 250 |
| ... | ... | St. George and St. James ... ... ... | ... | 2,004 | 548 | 642 | 9 | 28 | 679 | ... |
| ... | ... | St. George's, Hanover Square ... ... | ... | 726 | 495 | 386 | ... | 132 | 518 | ... |
| ... | ... | Western ... ... ... ... ... ... | ... | 12,037 | 1,556 | 370 | 404 | 669 | 1,443 | ... |
| ... | ... | Westminster General ... ... ... ... | ... | 6,797 | 668 | 349 | 170 | 117 | 636 | 500 |
| 1,090 | 810 | | 10,317 | 168,415 | 116,304 | 66,984 | 12,201 | 13,411 | 92,596 | 45,666 |

### STRATFORD AND EAST END DISTRICT.

Comprising Bethnal Green, Tower Hamlets, West Ham, Whitechapel, Hackney, Stepney, Limehouse, Poplar, and the East.

| No. of Beds. | No. of Beds Daily occupied | Hospitals. | In-patnts. | Out-patients. | Total Expenditure. | Charitable. | Proprietary. | Patnts pymnts. | Total Income. | Legacies not included in previous column. |
|---|---|---|---|---|---|---|---|---|---|---|
| 130 | 112 | German ... ... ... ... ... ... | 1,515 | 22,828 | 9,940 | 5,673 | 2,215 | 514 | 8,402 | 1,508 |
| 776 | 659 | London ... ... ... ... ... ... | 12,603 | 189,638 | 90,740 | 39,996 | 25,077 | 1,447 | 66,520 | 27,158 |
| 50 | 39 | Mildmay Mission Hospital ... ... ... | 519 | 7,323 | 3,301 | 2,614 | 569 | 42 | 3,225 | 200 |
| 71 | 56 | Poplar ... ... ... ... ... ... | 877 | 22,072 | 7,595 | 7,800 | 946 | 122 | 8,868 | 2,142 |
| 60 | 42 | West Ham, &c. ... ... ... ... ... | 588 | 20,546 | 4,729 | 4,804 | 194 | ... | 4,998 | 19 |
| 34 | 23 | Walthamstow, &c. ... ... ... ... | 443 | 1,000 | 1,147 | 1,534 | 239 | ... | 1,773 | ... |
| 164 | 103 | City of London for Diseases of the Chest ... | 800 | 9,169 | 11,026 | 9,338 | 270 | ... | 9,608 | 3,230 |
| 131 | 106 | East London for Children ... ... ... | 1,763 | 35,585 | 10,417 | 7,107 | 1,192 | ... | 8,299 | 1,610 |
| 38 | 35 | St. Mary's, Plaistow ... ... ... ... | 569 | 14,238 | 3,330 | 3,513 | 197 | 98 | 3,808 | ... |
| 18 | 12 | East End Mothers' Home ... ... ... | 264 | 255 | 1,479 | 1,053 | 478 | 46 | 1,577 | ... |
| 8 | 7 | Passmore Edwards Cottage, Tilbury | 81 | 401 | 664 | 701 | 22 | 3 | 726 | ... |
| 1,480 | 1,194 | DISPENSARIES. | 20,022 | 323,055 | 144,368 | 84,133 | 31,399 | 2,272 | 117,804 | 35,867 |
| ... | ... | Eastern ... ... ... ... ... ... | ... | 5,535 | 757 | 281 | 273 | 62 | 616 | 100 |
| ... | ... | Hackney Provident ... ... ... ... | ... | 928 | 247 | 63 | ... | 197 | 260 | ... |
| ... | ... | London ... ... ... ... ... ... | ... | 2,183 | 443 | 141 | 281 | ... | 422 | ... |
| ... | ... | Queen Adelaide's ... ... ... ... | ... | 6,078 | 510 | 281 | 220 | 39 | 540 | 113 |
| ... | ... | Tower Hamlets ... ... ... ... ... | ... | 4,142 | 619 | 377 | 22 | 167 | 566 | ... |
| ... | ... | Whitechapel Provident ... ... ... ... | ... | 5,048 | 873 | 72 | ... | 795 | 867 | ... |
| 1,480 | 1,194 | | 20,022 | 346,969 | 147,817 | 85,348 | 32,195 | 3,532 | 121,075 | 36,080 |

### THE METROPOLITAN HOSPITALS.—A SUMMARY OF THE WORK DONE IN 1899.

It will be seen from the following summary that the Voluntary Hospitals and Medical Charities of London, during the twelve months ending 31st December, 1899, relieved over One million seven hundred thousand patients at a cost of £911,589. The Ordinary Income only amounted to £707,362, leaving a deficiency of £204,227 on the year's work. The Legacies received in 1899 amounted to £279,176, being £16,735 more than the amount received in 1898.

| No. of Beds. | No. of Beds Daily Occupied. | Hospitals and Dispensaries. | In-patients. | Out-patients. | Total Expenditure. | Income. | | | Total Income. | Legacies not included in previous column. |
|---|---|---|---|---|---|---|---|---|---|---|
| | | | | | | Charitable. | Proprietary. | Patients' Payments. | | |
| | | | | | £ | £ | £ | £ | £ | £ |
| 1,906 | 1,406 | Newington and South District ... | 20,439 | 336,692 | 202,294 | 50,449 | 94,979 | 16,149 | 161,577 | 69,314 |
| 675 | 456 | City and East Central District ... | 7,666 | 194,194 | 65,543 | 35,308 | 8,427 | 5,642 | 49,377 | 13,821 |
| 1,090 | 810 | Westminster District ... ... | 10,317 | 168,415 | 116,304 | 66,984 | 12,201 | 13,411 | 92,596 | 45,666 |
| 1,508 | 1,169 | St. Marylebone and West Central District ... ... ... ... | 14,426 | 230,788 | 127,010 | 71,553 | 22,461 | 12,055 | 106,069 | 31,464 |
| 1,684 | 1,326 | Kensington and West District ... | 15,231 | 169,833 | 166,995 | 67,223 | 30,861 | 7,710 | 105,794 | 53,235 |
| 1,084 | 764 | Islington and North-West District | 9,632 | 157,987 | 85,626 | 52,837 | 10,467 | 7,570 | 70,874 | 29,596 |
| 1,480 | 1,194 | Stratford and East-End District... | 20,022 | 346,969 | 147,817 | 85,348 | 32,195 | 3,532 | 121,075 | 36,080 |
| 9,427 | 7,125 | | 97,733 | 1,604,878 | 911,589 | 429,702 | 211,591 | 66,069 | 707,362 | 279,176 |

### HOW TO GIVE.

ENOUGH has been said to show how great are the claims of the hospitals; how real is the good they are doing; how pitiable is the condition of those whom they relieve; how hopeless would be the lot of those unhappy ones if the hospitals did not exist; how essential a part hospitals play in the social fabric of our great cities; how they stand ever before the world as a proof of the sympathy existing between the strong and the weak; and how, if we have in us a spark of that sympathy with suffering which is one of the highest attributes of humanity, we ought every one of us to make an effort, a personal and individual effort, to help the hospitals in their noble work.

How is this to be done? Perhaps the best way would be to interest oneself in a particular hospital, to find out its wants, and do our best to meet them. There is no fear that such a course would result in one's finding that such an institution had no wants, or that we could do nothing for it. Every hospital wants a great deal more than it gets. There is always room for gifts of flowers, fruit, game, old linen, and the hundred and one odd things which add to the brightness and take somewhat from the misery of the sufferers in its wards, but what every hospital wants first and above all things is money. Money for the food, the physic, the warmth the bedding, the nursing, the comforts, the cleanliness, and the elaborate methods of treatment which make recovery from illness so different in a hospital to what it is in the dwellings of the poor.

If any one will visit the sick poor in their own homes, especially those of them who have not the benefit of the ministrations of a district nurse, and then visit them in a hospital, he will see at a glance how much hospitals do to minimise suffering and help the regaining of health. He may, perhaps, think the directors are wrong in this point or that; but he will see that no defects in our hospital system can hide the main fact hat hospitals are an inestimable boon to the sick and suffering. And he will see something more, for what, above all things, will be forced in upon him, is that all the appliances and all the methods which so strongly make for health cost money. Thus surely he will be more inclined to put his hand in his pocket and assist in the good work.

But we cannot all find either time or opportunity to visit hospitals. Nor can one desire that a hospital ward should be made a public show. We might, perhaps, read hospital reports; and a perusal of such reports would have a wonderful effect in opening the eyes of any one who wants to know "what the hospitals do with all the money they get." It is to be feared, however, that we cannot all of us even read hospital reports. We know the hospitals exist, we know what good they are doing, we know that they must at any price be carried on, and we can guess how enormous is the cost of keeping up such great establishments, but of the details most of us can know nothing. Even how to give is with some a difficulty. How to give? How to assist this great work? How to choose what hospital to help? How to know which hospital is in most urgent want, and which hospital will make best use of our money? These are questions which embarrass some people, and which with others turn themselves into excuses for not giving at all, and here it is that the Hospital Sunday Fund comes in so usefully.

Hospital Sunday comes but once a year, and surely none should miss the opportunity which it offers of helping the hospitals. No one sees the gift, no one can say whether it is much or little—for often people give nothing because they cannot give what their neighbours think they ought to afford—no one but Him, in Whose Name and for Whose Sake it is given. It will be wisely expended; the men who compose the Hospital Sunday Committee are men of tried probity and honour, men of tried wisdom and discretion. They divide the people's offerings carefully, with due respect to the work done by each institution, and the income available for that work. Every hospital must depend for the most part on the voluntary contributions which it receives from year to year. Not all can pledge themselves to give a fixed sum every year, for not all have a fixed and secure income. But all can give something. Those who have given already will, we may be sure, put something into the offertory bag on Hospital Sunday, for those who have given are the most willing to give. But those who have not given should now begin to do so, and should take this annual opportunity of helping those who are poor, helpless, and suffering.

## Figures and Tables

**Figure f1:**
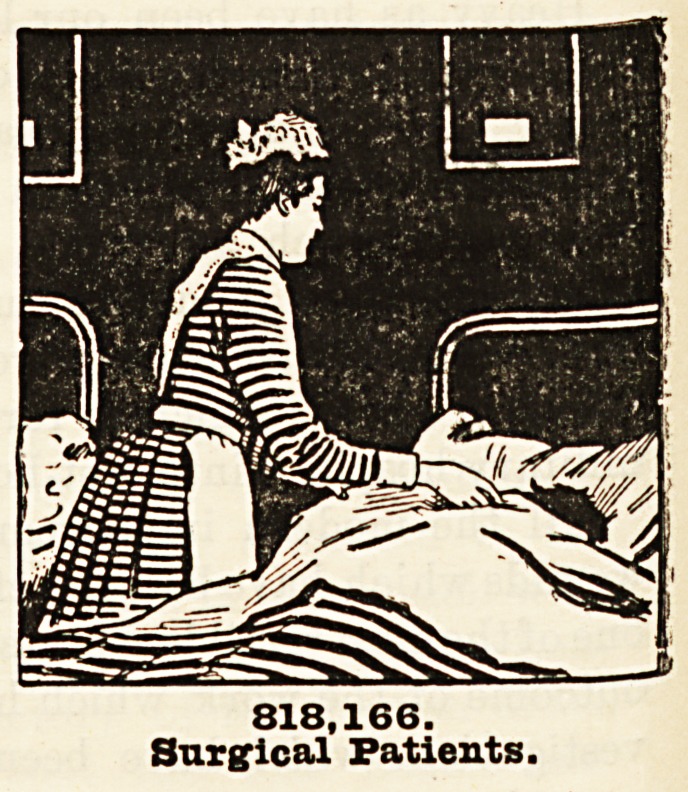


**Figure f2:**
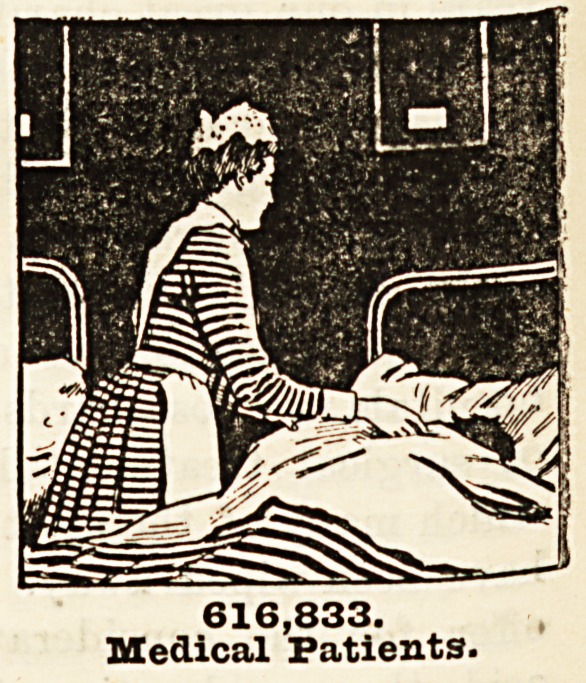


**Figure f3:**
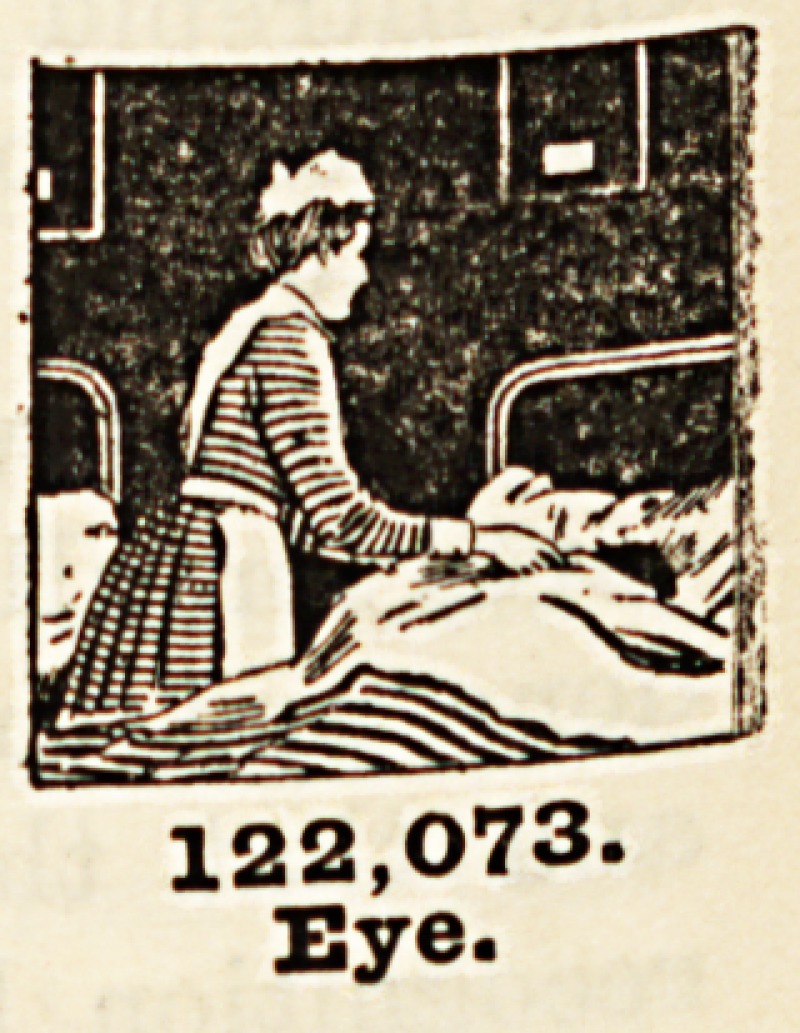


**Figure f4:**
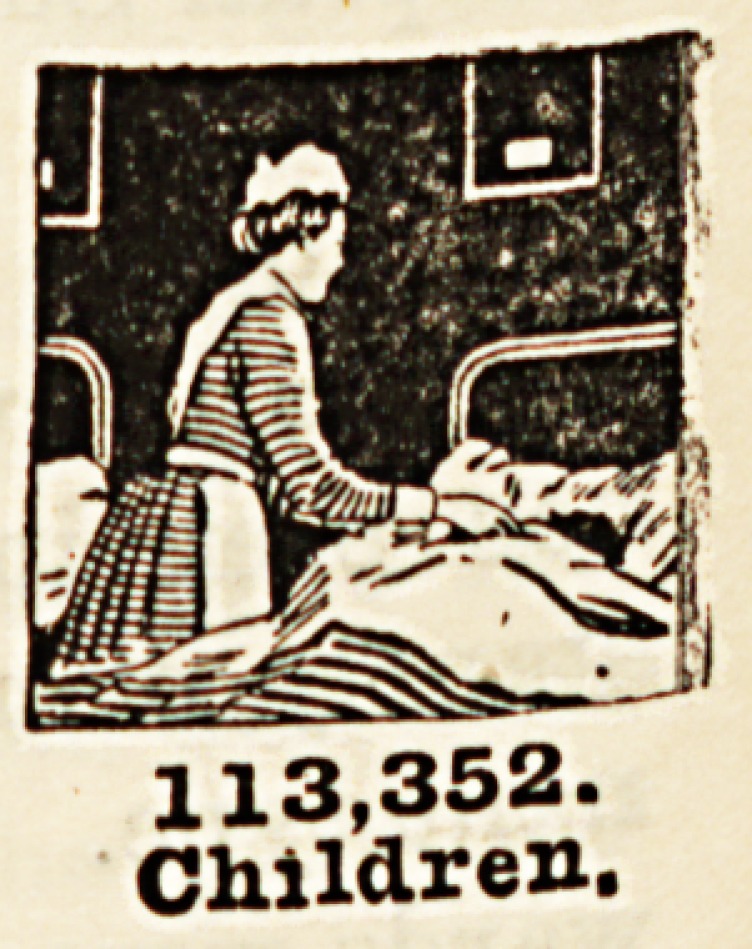


**Figure f5:**
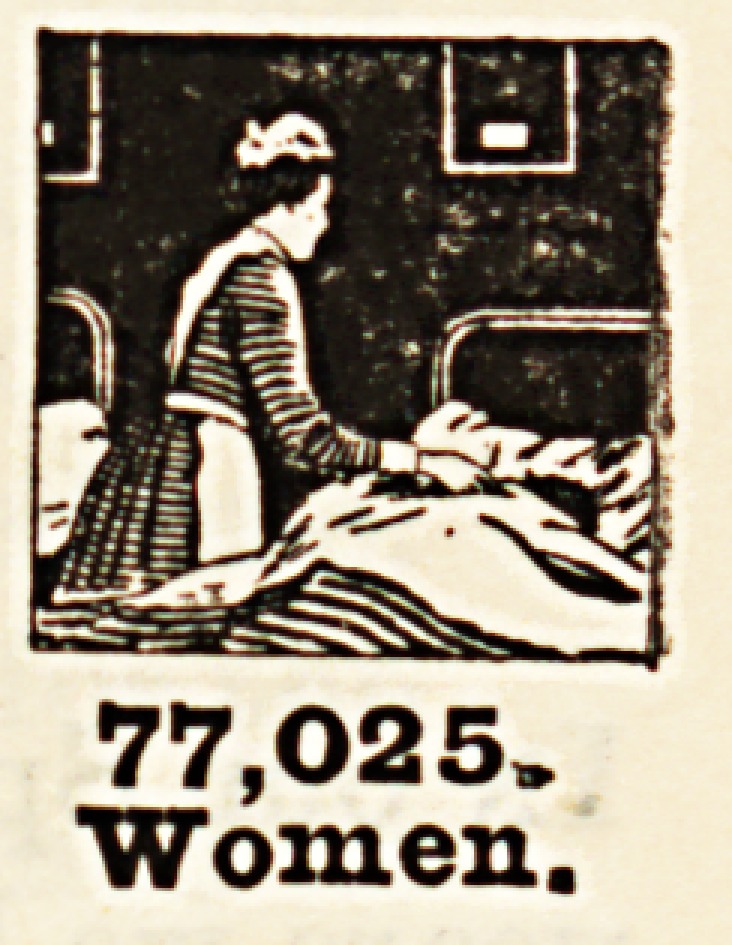


**Figure f6:**
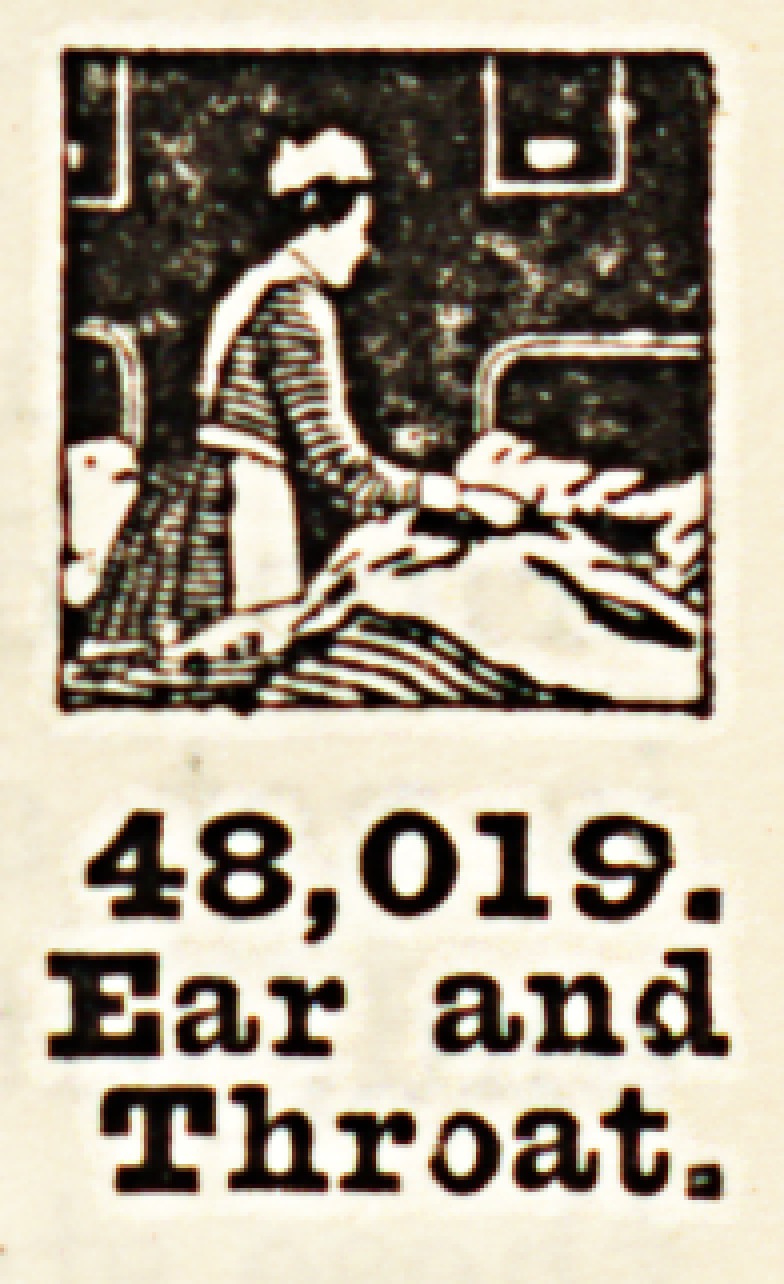


**Figure f7:**
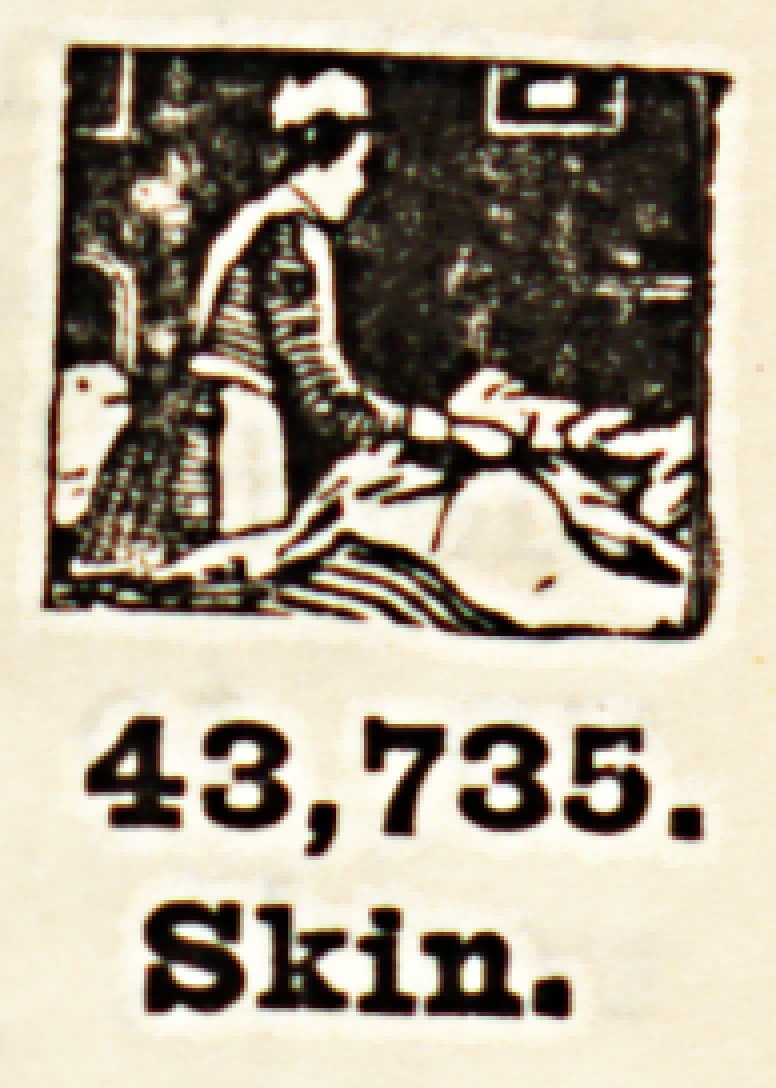


**Figure f8:**
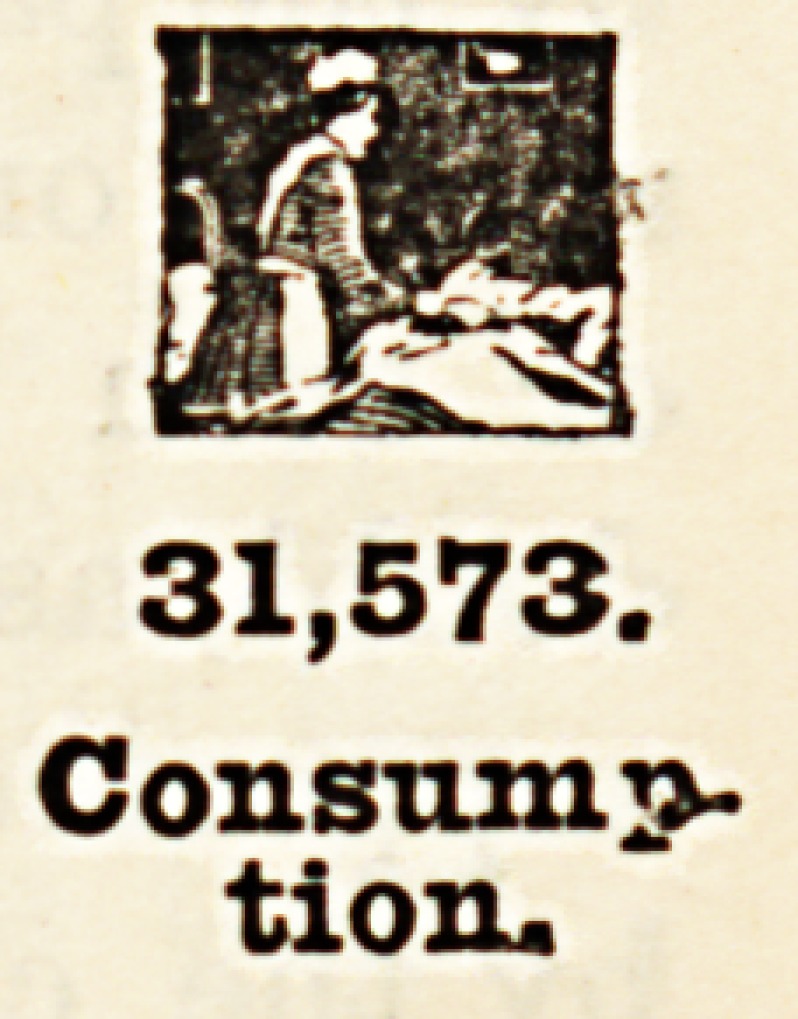


**Figure f9:**
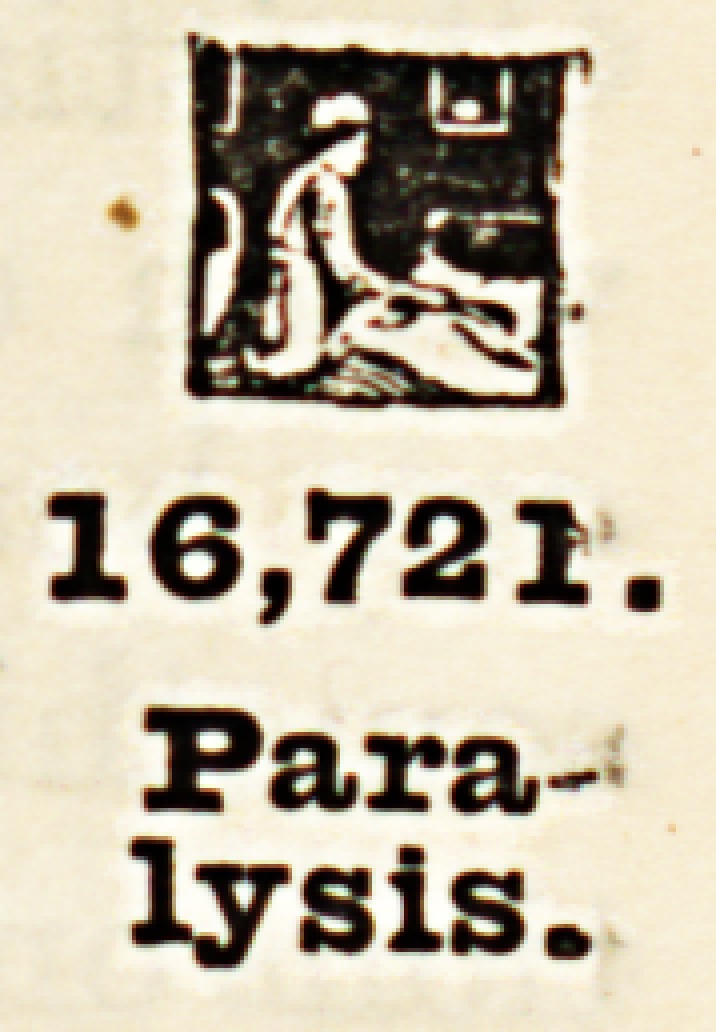


**Figure f10:**
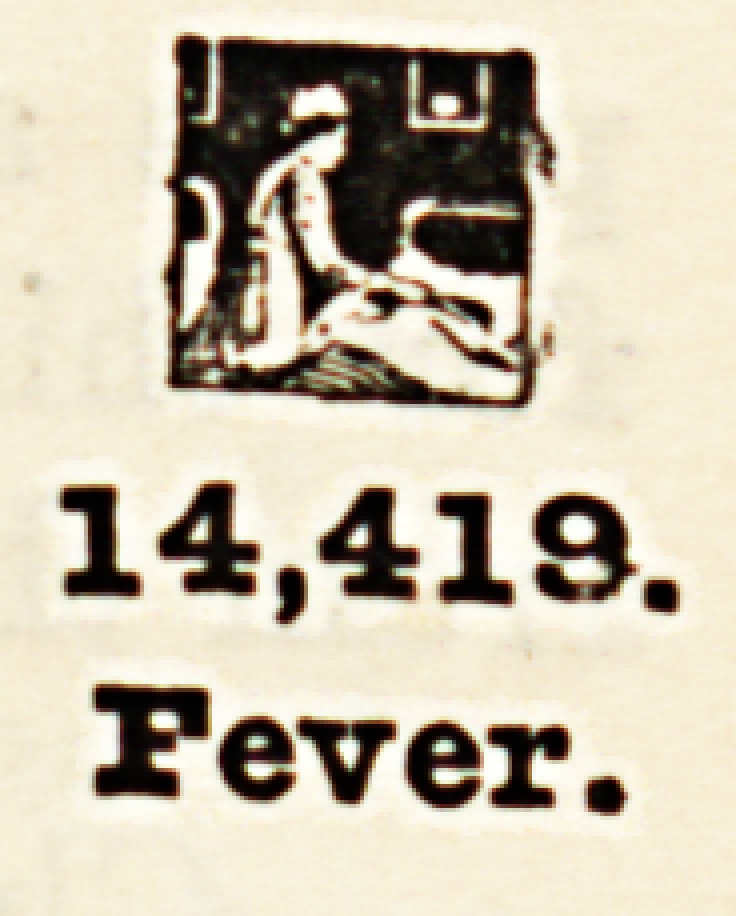


**Figure f11:**
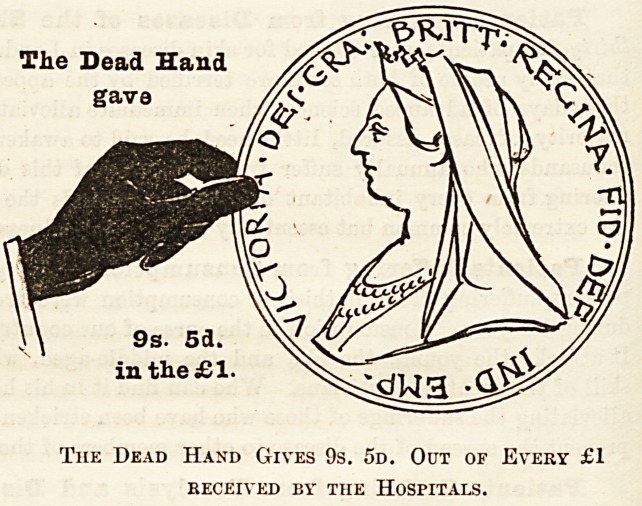


**Figure f12:**
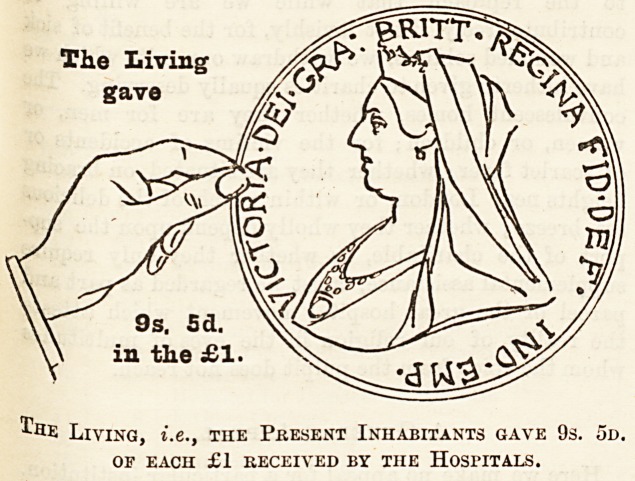


**Figure f13:**